# Complications of Microsurgery of Vestibular Schwannoma

**DOI:** 10.1155/2014/315952

**Published:** 2014-05-28

**Authors:** Jan Betka, Eduard Zvěřina, Zuzana Balogová, Oliver Profant, Jiří Skřivan, Josef Kraus, Jiří Lisý, Josef Syka, Martin Chovanec

**Affiliations:** ^1^Department of Otorhinolaryngology, Head and Neck Surgery, 1st Faculty of Medicine, Faculty Hospital Motol, Charles University in Prague, V Uvalu 84, Prague 5, 150 06 Prague, Czech Republic; ^2^Department of Auditory Neuroscience, Institute of Experimental Medicine, Academy of Sciences of the Czech Republic, Videnska 1083, Prague 4, 142 20 Prague, Czech Republic; ^3^Department of Pediatric Neurology, 2nd Faculty of Medicine, Faculty Hospital Motol, Charles University in Prague, V Uvalu 84, Prague 5, 150 06 Prague, Czech Republic; ^4^Department of Imaging Methods, 2nd Faculty of Medicine, Faculty Hospital Motol, Charles University, V Uvalu 84, Prague 5 150 06, Prague, Czech Republic

## Abstract

*Background*. The aim of this study was to analyze complications of vestibular schwannoma (VS) microsurgery. *Material and Methods*. A retrospective study was performed in 333 patients with unilateral vestibular schwannoma indicated for surgical treatment between January 1997 and December 2012. Postoperative complications were assessed immediately after VS surgery as well as during outpatient followup. *Results*. In all 333 patients microsurgical vestibular schwannoma (Koos grade 1: 12, grade 2: 34, grade 3: 62, and grade 4: 225) removal was performed. The main neurological complication was facial nerve dysfunction. The intermediate and poor function (HB III–VI) was observed in 124 cases (45%) immediately after surgery and in 104 cases (33%) on the last followup. We encountered disordered vestibular compensation in 13%, permanent trigeminal nerve dysfunction in 1%, and transient lower cranial nerves (IX–XI) deficit in 6%. Nonneurological complications included CSF leakage in 63% (lateral/medial variant: 99/1%), headache in 9%, and intracerebral hemorrhage in 5%. We did not encounter any case of meningitis. *Conclusions*. Our study demonstrates that despite the benefits of advanced high-tech equipment, refined microsurgical instruments, and highly developed neuroimaging technologies, there are still various and significant complications associated with vestibular schwannomas microsurgery.

## 1. Introduction

Vestibular schwannomas (VS) are the most common tumors of the cerebellopontine angle (CPA; 80–95%). They are benign, slow growing tumors arising from the Schwann cells at the oligodendrocyte-Schwann cell junction (Obersteiner-Redlich zone). These tumors originate from the peripheral portion of the inferior and superior vestibular nerve and very rarely from the cochlear branch of the eighth cranial nerve. VS are rare, comprising approximately 10% of primary intracranial tumors, 85% of CPA tumors, and 90% of intracranial schwannomas [1].

The symptoms of VS include mainly sensorineural hearing loss (SNHL), tinnitus, and vestibular disorder and rarely other cranial nerve lesions and intracranial hypertension. Treatment options of VS depend on several factors (size and growth of the tumor, symptoms, and medical comorbidities) and require an active approach (surgical removal; stereotactic radiosurgery) or observation (wait and scan). VS represent histologically benign tumours, a significant proportion of which are either nongrowing or slow-growing; therefore, observation is commonly accepted option in case of small oligosymptomatic tumors (<2.5 cm in size) [2, 3]. Preservation of neurological function and elimination of complications is a principal goal of any therapeutic action. Although microsurgery represents a gold standard in VS management, there are strong proponents of stereoradiosurgical treatment for tumors ≤2.5–3 cm in diameter [4, 5]. Microsurgery is indicated mainly in the case of large tumors, the deterioration of useful hearing during observation with attempt for its preservation, and in the case of disabling symptoms [2, 3].

Removal of vestibular schwannomas may be performed using several approaches: the translabyrinthine [6], the retrosigmoid [7], or the middle fossa approach [8]. Prerequisites for the translabyrinthine approach include mainly the preoperative lack of serviceable hearing and the presence of larger tumors [9, 10]. The retrosigmoid (suboccipital) approach allows the removal of tumors of any size with the potential for hearing preservation; its main disadvantage is the need for cerebellar retraction [11, 12]. Lastly, the middle fossa approach is chosen for tumors limited to the internal auditory canal or with minimal CPA extension with an attempt to preserve hearing. Drawbacks of this approach are the limited access to CPA and the need for temporal lobe retraction [8].

Over the past several decades, the outcomes of treatment for patients with VS have significantly improved. The goal of the treatment in the early 20th century was to resect the tumor without perioperative mortality. However, with the improvement of diagnostic tools (early stage diagnosis), advances in anesthetic care, introduction of microsurgical techniques, and intraoperative monitoring, mortality and neurologic morbidity have been significantly reduced without compromising the radicality of tumor resection. Therefore, it became possible to preserve normal function of cranial nerves, including facial nerve and hearing preservation, whilst causing minimal injury to the cerebellum and brainstem.

Early diagnosis, advanced skull base surgery techniques, specific surgical approaches, tumor size, age of the patient, and the use of intraoperative monitoring have all been implicated as predictive factors for good postoperative recovery with a decreased complication rate [13]. However, complications may still occur, especially when treating large tumors. Precise information about these potential complications has to be given to the patient at the time of the surgical decision. There is also imminent need of their effective diagnosis, management, and prevention. The most common complications of VS microsurgery are CSF leak and meningitis, facial nerve paresis, headache, disordered vestibular compensation, cerebellar and brain stem injuries, and vascular complications [2, 3, 14–20].

Based on recent trends our goal was to analyze the complications encountered in a series of 333 consecutive patients undergoing microsurgical treatment over the last 15-year period. We also reviewed the international literature on the complications of vestibular schwannoma microsurgery and its prevention and management.

## 2. Material and Methods 

A retrospective study was performed on 333 patients, with ages ranging from 12 to 74 years (48 ± 14 years): 144 male (43%) and 189 female (57%) patients with unilateral vestibular schwannoma indicated for surgical treatment based on a retrosigmoid-transmeatal approach, across the period between January 1997 and December 2012. All patients were operated on by the same team of neurotologists and a neurosurgeon. The design of the study was approved by the local ethical committee.

The data collected in each patient included the patient's age, gender, size of tumor, intraoperative findings (e.g., facial nerve structural and functional preservation and radicality of tumor resection), and postoperative complications (CSF leak, meningitis, vascular complications, headache, cranial nerve dysfunction, and altered vestibular compensation).

The size of the tumor was determined by preoperative magnetic resonance imaging (MRI). The diameter was measured from the extrameatal component on the axial scans in the plane parallel with the long axis of the internal auditory canal (IAC), which included both intra- and extrameatal portion of the tumor. Koos grading system was also used to classify the tumor grade based on tumor extension (G1: intrameatal tumors; G2: tumors extending to the cerebellopontine angle; G3: tumors filling the cerebellopontine angle; G4: tumors compressing the brainstem and cerebellum).

Facial nerve function was assessed according to the House-Brackmann (HB) grading system immediately after surgery and at the time of the last followup. Function was further classified into three categories: excellent (HB I-II), intermediate (HB III-IV), and poor (HB V-VI). Facial nerve monitoring was used to identify the facial nerve and confirm its function intraoperatively in all cases.

Postoperative complications were evaluated during the immediate postoperative recovery (within one week) and at the time of last followup (long-term or persistent complications). The follow-up period ranged from 12 to 178 months. Symptoms of vertigo, spontaneous nystagmus, and deviation of subjective visual vertical, which were persistent after six months following operation, were classified as disordered compensation of vestibular pathology.

## 3. Results

Primary microsurgical vestibular schwannoma removal was performed in 317 patients. Seven patients underwent revision surgery because of a growing tumor remnant following a primary surgery performed elsewhere. Five patients were operated on because of a growing tumor after previous stereoradiosurgical treatment (three patients were treated with Leksell's gamma knife and 1 with LINAC) and another four patients because of a growing tumor after previous partial tumor resection followed by stereoradiosurgery (all were treated with Leksell's gamma knife (LGK)). We employed the retrosigmoid-transmeatal approach in 325 (97%), translabyrinthine approach in six (2%), and combined translabyrinthine-retrosigmoid approach in two (1%) of the cases.

In our series grade one tumors were present in 12 patients (3.6%), grade two in 34 patients (10.2%), grade three in 62 patients (18.6%), and grade four in 225 patients (67.6%). In nine (3%) patients tumors caused hydrocephalus and intracranial hypertension. We would like to stress here the atypically high proportion of the grade four tumors.

Gross total removal (GTR) of tumors was achieved in 328 (98.5%) of cases. In five patients we decided to perform near total tumor removal (NTR) leaving residual tumor capsules of ≤1-2 mm either at the root exit zone of CN VII (as in one case) or at CN VII in the region of porus of IAC (four cases). We did not observe any tumor regrowth on the repeated annual MRI scans. We encountered only two cases of tumor recurrence in the group of GTR (one in the region of the fundus of IAC and one in the labyrinth extending to the IAC).

CN VII function preservation noted immediately after surgery was deemed “excellent” in 175 patients (55%), “intermediate” in 96 patients (30%), and “poor” in 48 patients (15%). CN VII function at the last followup was “excellent” in 214 patients (67%), “intermediate” in 98 patients (31%), and “poor” in 15 patients (2%). Overall facial nerve injury leading to discontinuation was present in 22 patients (6.6%) after primary surgery and in all but one patient after previous primary stereoradiosurgery or stereoradiosurgery preceded by partial tumor resection (PTR) (Table 1). In all such cases we performed facial nerve reconstruction. Direct neurorrhaphy in the IAC without grafting was possible in only two patients. In 12 patients we performed CN VII-VII anastomosis with grafting in the IAC/CPA, employing the graft from the greater auricular nerve, and in four patients we performed extra-intracranial CN VII-VII anastomosis according to the Norman-Dott method, using the sural nerve graft. In one patient we employed transpetrous CN VII-VII anastomosis with sural nerve according to Samii. Cross-anastomosis CN VII–CN XII end to side according to the technique described by Darrouzet et al. [21] was employed in cases of proximal stump of CN VII absence (two patients from the group of primary microsurgery and nine patients in the group of revision microsurgery following previous stereoradiosurgery and eventual PTR) and in one patient with preserved facial nerve following previous LGK treatment, but the patient had persistent HB VI function even after 18 months postoperatively without any sign of ongoing reinnervation based on electromyography (Table 2).

Transient postoperative dysfunction of the trigeminal nerve was observed in seven patients (2%) and the permanent lesion in three patients (1%). One patient (0.3%) had an iatrogenic lesion of the CN VI after accidental VII-VI anastomosis. Transient postoperative dysfunction of lower cranial nerves (CN IX–XI) occurred in 20 patients (6%) with giant VS (>4 cm extrameatal tumor component).

Disordered vestibular compensation was observed in 43 patients (13%).

**Figure 1:**
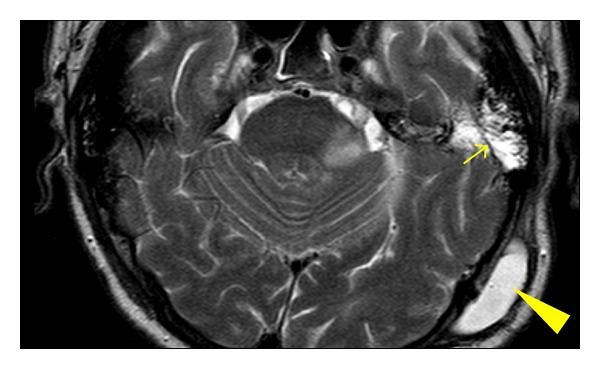
Lateral variant of CSF leak/pseudomeningocele (T2W MRI). Arrowhead: pseudomeningocele, arrow demonstrates CSF filled pneumatic system of the temporal bone.

**Figure 2:**
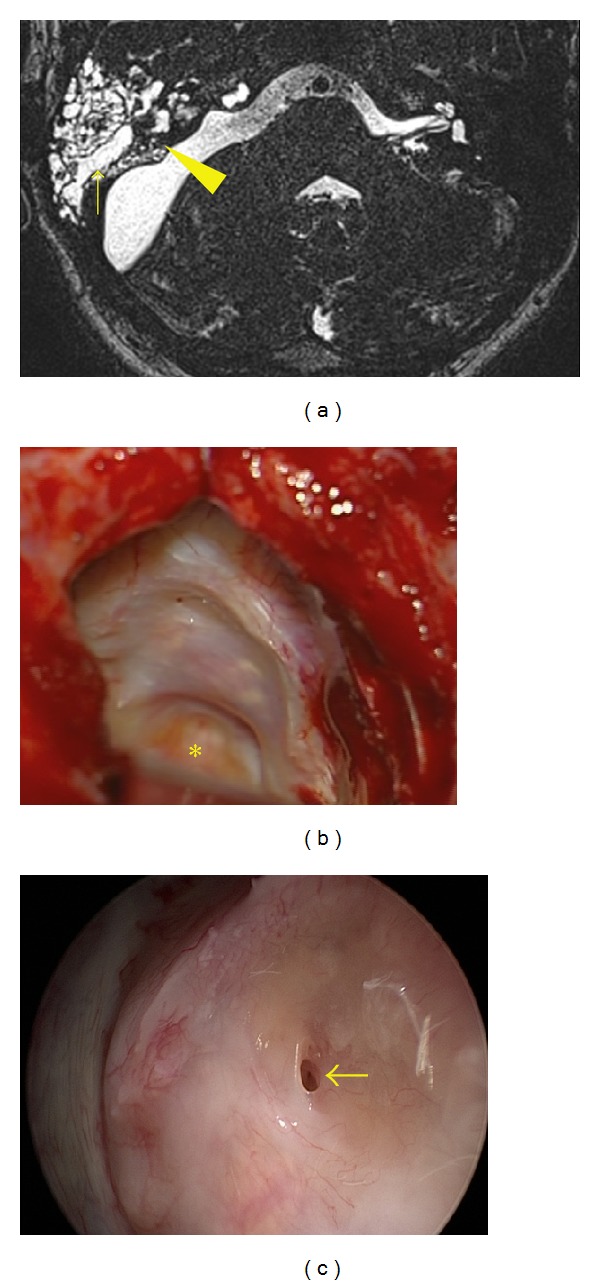
Medial variant of CSF leak. (a) T2W MRI, arrowhead pointing to the fistula; arrow demonstrates CSF filled pneumatic system of temporal bone. (b) Wound revision with identification of fistula in the posterior rim of meatotomy; asterisk shows the closed IAC. (c) Endoscopic view of the fistula.

A lateral variant of CSF leakage (pseudomeningocele) (Figure 1) occurred in 208 patients (62.5%). In 205 patients it was managed conservatively using a puncture, aspiration, with or without tissue glue injection (84 patients, one to six applications), and wound compression. A medial variant of CSF leakage (Figure 2) occurred in two patients (0.6%) and was managed by wound revision and leak sealing. During the follow-up period (1–15 years in all 333 patients after surgery), neither infection nor meningitis occurred.

Postoperative headache was reported by 29 patients (9%).

**Figure 3:**
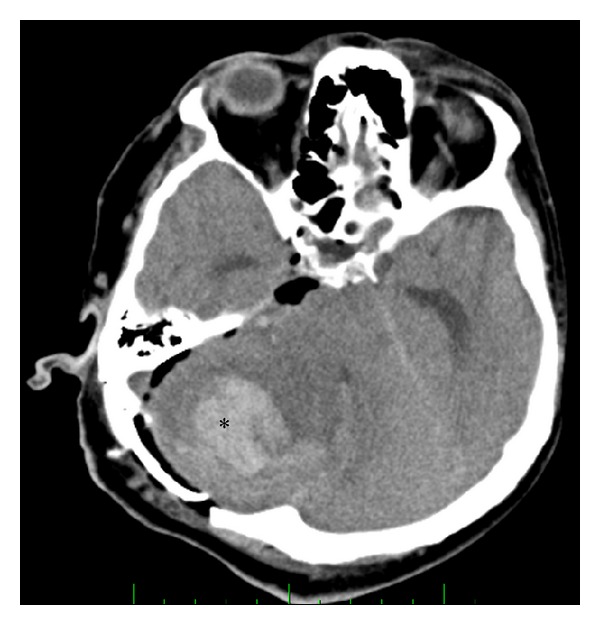
CT scan of patient with intracerebellar haematoma.

**Figure 4:**
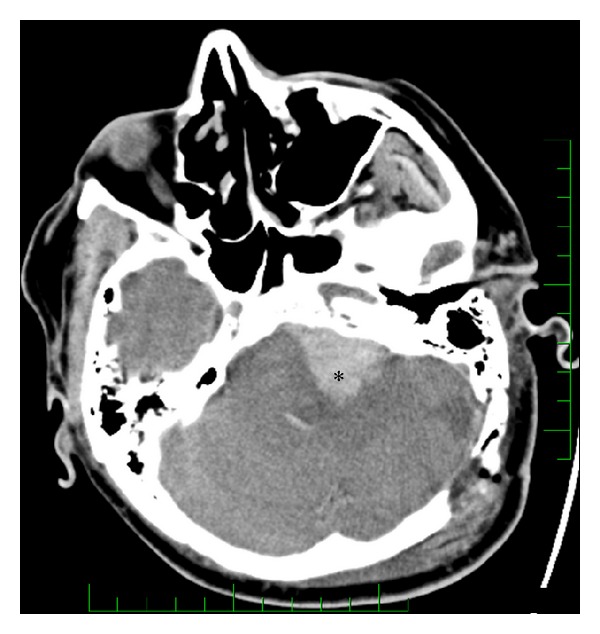
CT scan of patient with haematoma of the cerebellopontine angle.

**Figure 5:**
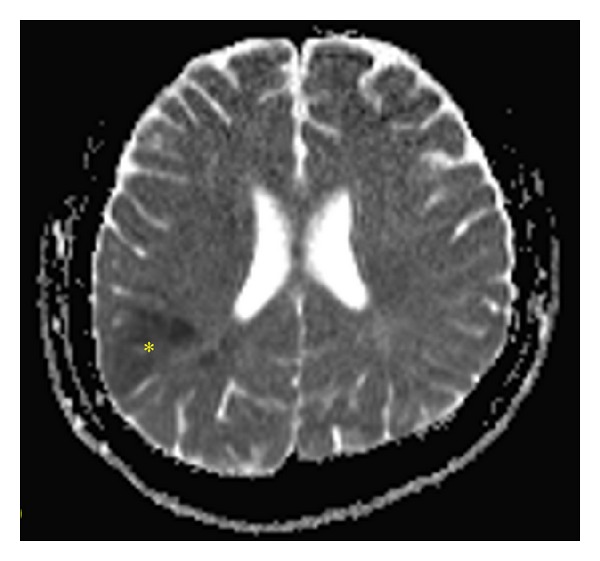
MRI of patient with the supratentorial ischemia as a consequence of microembolisation (paradox embolisation excluded).

**Figure 6:**
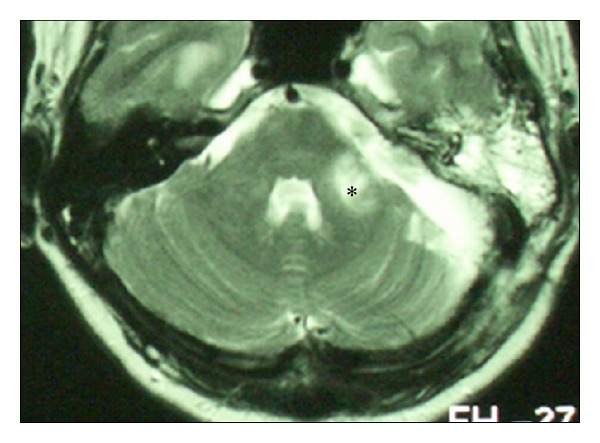
MRI of patient with peduncular venous infarction (asterisk) due to superior petrous vein injury.

Hemorrhage after VS microsurgery occurred in 15 patients (5%). Intracerebellar haematomas (Figure 3) were observed in four patients (1.2%) and CPA haematomas (Figure 4) in eight patients (2.4%). All of these were managed with immediate wound revision and decompression. We also encountered epidural haematoma in three patients (1%). Two of these epidural haematomas were managed with wound revision and one case with puncture and aspiration only. In one patient supratentorial ischemia caused by microembolization (Figure 5) occurred and one patient suffered peduncular venous infarction caused by superior petrous vein injury (Figure 6). Both of them suffered from transient organic psychosyndrome.

The mortality rate in our study was approximately 3%. The cause of death in two patients was intracerebellar haemorrhage and pulmonary embolism in one case.

We also encountered one case of myelopathy related to cervical disc herniation as a consequence of unexpected intraoperative patient arousal from general anaesthesia. Other rare nonsurgical complications, for example, displacement of central venous catheter, phlebitis, transitory lesion of the ulnar (two cases), and peroneal (one case) nerve, were also observed.

Postoperative hearing loss/deafness was not considered a complication; therefore, it is not evaluated in this series.

## 4. Discussion

Morbidity and mortality rates associated with the surgical treatment of vestibular schwannomas have changed significantly during the past century. In the early 1900s Harvey Cushing developed techniques to reduce the surgical mortality rate from 80% to 20% [20, 22–24] and during the 1960s major advances in anaesthesia, pharmacology, and especially surgical techniques were developed to a large extent by William House [22]. As mortality and morbidity have been reduced dramatically in the recent years, preservation of quality of life postoperatively has become the generally accepted goal of VS management.

In the case of microsurgical treatment, gross total tumor removal with the preservation of neurologic functions is the newest benchmark. Despite this some works support near total and subtotal removal in large tumors as suitably viable treatment options to maintain good postoperative facial nerve and even hearing functions [14, 25].

The reduced complication rate is a result of the introduction of modern methods and materials into the surgical treatment of VS. In general, the increased visualization of the surgical field, through the use of endoscopes, helps to identify possible CSF leakage and also decreases the chance of residual tumor, especially intrameatally. Faster tumor removal is managed by the use of ultrasonic aspirators, shavers, or fibre lasers. The position of craniotomy according to sigmoid sinus and avoidance of its injury can be improved by CT navigation. Diffusion tensor imaging (DTI) of facial nerves helps in easier identification of its course. New materials used for dura mater and skull reconstruction should help to prevent CSF leakage as well as the adhesion of nuchal muscles that cause headaches.

The most common complications of microsurgical treatment are CSF leak and meningitis, facial paresis, headache, vestibular disorders, cerebellar and brain stem injuries, and vascular complications. These postoperative complications can be divided into two groups: neurological and nonneurological.

### 4.1. Nonneurological Complications

#### 4.1.1. CSF Leak and Meningitis

CSF leaks and meningitis are the most common complications following vestibular schwannoma resection. The literature reports a range of postoperative CSF leak rates from 2% to 30% [26–29] but the average leak rate appears to be approximately 10% [30].

CSF leakage can be classified as either of a medial (via petrous air cells or eventual labyrinth) or lateral variant (wound leak/pseudomeningocele). The most difficult to manage are the medial variants that usually require revision surgery and leak closure.

#### 4.1.2. Medial CSF Leak

Many reports have discussed different factors leading to postoperative CSF leaks. Both Slattery et al. [20] and Brennan et al. [31] found a significant relationship between tumor size and the prevalence of CSF leakage; according to Brennan et al., larger tumors appeared to lead to a greater risk of CSF leak; however, Slattery et al. [20] showed a correlation between surgical approach and CSF leakage rate, with a retrosigmoid approach having the highest frequency (15%) and middle fossa approach the lowest (5.7%). Lüdemann et al. [32] observed that only large tumors with severe dislocation of the brainstem, causing hydrocephalus, showed a higher incidence of CSF; otherwise, they showed inverse correlation (smaller tumors had a higher risk of CSF leak). Sanna et al. [15] did not demonstrate any relationship and medial CSF leak in the case of the translabyrinthine approach. Based on these findings, it can be concluded that tumor size and type of surgical approach are the main factors affecting postoperative CSF leakage.

Merkus et al. [33] in their long term study of 1803 cases operated via translabyrinthine approach reported only 0.8% of postoperative CSF leaks and stressed meticulous sealing of petrous bone air cells. The same is true for the retrosigmoid-transmeatal approach in which the most common pathways of leak formation are perimeatal air cells of petrous bone; therefore, their sealing is crucial for CSF leak prevention. The addition of endoscopic and endoscopy-assisted vestibular schwannoma surgery seems to be beneficial for improved identification of potential pathways of CSF leakage [34]. The material used for petrous bone air cells sealing can also be considered a significant factor. The work of Lüdemann et al. [32] supports a fat implantation as superior to muscle implantation for the prevention of CSF leakage. Their study on 420 patients undergoing tumor removal via retrosigmoid-transmeatal approach reports the incidence of postoperative CSF leaks at 2.2% with the use of fat implantation compared to 5.7% if muscle grafts were employed for petrous air cell sealing. Furthermore, women had less postoperative CSF leakage (3.4%) than men (5.6%).

Our results show that with meticulous sealing of opened air cells following IAC opening, medial variant of CSF leakage can be reduced to a minimum even in cases with high proportions of large tumors. We have observed only two such cases and both of them were managed surgically. Suspicion can be based on intraoperative findings (e.g., significant perimeatal pneumatisation) and the absence of pseudomeningocele. MRI can be helpful in confirming and localizing the pathologic pathway. Despite the majority of experts recommending the horizontal positioning of patient with elevated head and lumbar drainage for cases of leakage persisting for more than 24 hours, we are proponents of early leak sealing as the first leaving surgical revision for cases of leaks persisting more than three days.

#### 4.1.3. Lateral CSF Leak

The study by Mangus et al. [35] showed a correlation between the surgical approach and type of CSF leak [26], in which wound leak was most commonly present in the use of translabyrinthine (54%), whereas rhinorrhea was most common in the cases of the suboccipital (68%) and middle fossa (70%) approach. Incidence of wound leak in our study was much higher than in the published studies. This could be related to the technique of wound closure, avoidance of lumbar drainage in the perioperative period, and our active approach in identifying wound leak. Moreover, our results show that management of the lateral variant of CSF leak is relatively straightforward (sole puncture, aspiration, and wound compression and in cases of recurrence the application of tissue glue). Wound revision for CSF leak was needed in less than 1% of all these cases when an anatomical obstacle preventing optimal closure of the dead space, created by bone and soft tissue removal, was identified intraoperatively (e.g., bony overhang over the dura mater).

Our recommendation for wound leak prevention is the use of the watertight multilayer-tissue closure technique, with a preferred primary suture of dura mater and the use of muscle, fat, tissue glue, and pressure dressing for several days. Under such conditions, we did not observe any prolonged wound healing or postoperative wound infection; however, our frequency rate of CSF wound leak is relatively high.

#### 4.1.4. Meningitis

Postoperative meningitis is a well described complication of skull based surgery. Although mortality is rare [15–17, 20, 36], early diagnosis and treatment are important in the management of these patients. Postoperative CSF leak is associated with an increased risk of meningitis that is raised from 3% to 14% [30]. Allen et al. [37] also reported an increased risk associated with the suboccipital approach. In contrast, Selesnick et al. [30] reported that a CSF leak was not associated with surgical approach, although the meningitis was significantly associated with cerebrospinal fluid leak. However, Kourbeti et al. [38] reported that presence of CSF leak was not significant in developing of meningitis. In general, CSF leak seems to be an obvious risk factor of meningitis.

Despite the high rate of the lateral variant of CSF leak in our series that were managed actively without lumbar drainage, we did not encounter neither wound infection nor meningitis. Thus, we can only speculate whether or not perioperative employment of lumbar drainage represents a risk of meningitis.

Our recommendations for the avoidance of meningitis are the prevention of wound contamination by the aseptic surgical field, perioperative antibiotic coverage, meticulous sealing of all potential pathways of CSF leaks, and active management of all types of CSF leaks.

#### 4.1.5. Headache

The incidence of chronic headache after VS surgery in the literature is widely variable ranging from 0 to 73% and depends on surgical approach as well as postoperative evaluation period [39–44]. The occurrence of postoperative headache has been reported mainly in patients undergoing tumor resection via the retrosigmoid approach [45]; however, the origin of postoperative headache in relation to surgical approach remains unclear. Many different factors may cause symptom: incision, dural adhesions to nuchal muscles or to subcutaneous tissues, dural tension, or muscle spasm [46]. A high-arching skin incision crossing the occipital nerves at its terminal branches is less likely to cause chronic postoperative headache compared to a straight incision. Schaller and Baumann [47] informed that the prevention of postoperative headache may include the replacement of the bone flap at the end of surgery, duraplasty instead of direct dural closure, and prevention of the use of fibrin glue. Catalano et al. [48] reported that free circulation of bone dust into the posterior fossa during intradural drilling of the IAC may be the most important factor in the development of headache after the retrosigmoid approach.

Our results fit to the published data with prevalence of postoperative headache being 9%. The vast majority of such cases were tension type headaches managed conservatively.

To prevent postoperative headache in our patients, we promote meticulous cleaning of the bone dust by continuous irrigation and suction, duraplasty as needed, cranioplasty to prevent dural adhesion to nuchal muscles, and proper placement of the incision [45]. In the case of postoperative headaches, multidisciplinary management in cooperation with a physiotherapist, neurologist, pain clinic, and even alternative approaches (e.g., acupuncture) are helpful.

#### 4.1.6. Intracranial Vascular Complications

Vascular complications of the VS surgery can have devastating consequences. These complications occur as an intracranial haemorrhage (intraparenchymal hematomas and subdural or epidural hematomas) or ischemia. Such complications can cause death if not immediately treated. Samii and Matthies [18] reported their acute and subacute postoperative haemorrhage cases as 2.2% and 1.5%, respectively. Sade et al. [49] concluded that the incidence of vascular complications in VS surgery was similar for retrosigmoid and translabyrinthine approaches (2.7%). The middle fossa approach has the highest occurrence of epidural haematomas.

The major consequences of the acute intracranial haemorrhage are mainly caused by increased intracranial pressure leading to loss of consciousness, hemiparesis, fixed or dilated pupil, respiratory distress, bradycardia, or systolic hypertension. The recognition of any of these signs should lead to early intervention. The majority of intracranial haemorrhages necessitate immediate wound revision following CT scanning.

Ischemic complications may be of arterial or venous origin and may affect the brain stem or the cerebellar hemisphere. Adhesion of the tumor with the brainstem and cerebellum is the cause of microtraumatism of the small vessels that course at the tumor-brain interface. Consequently, meticulous care to preserve the arachnoid plane during tumor removal is crucial for preservation of subpial vessels. Coagulation of small arterial perforators must be avoided to prevent brainstem infarction. Major injury of cerebellar vessels is rare because these vessels are usually well identified and safely dissected from the tumor. Care should also be taken to preserve large veins (e.g., greater petrosal vein) as their closure can have serious consequences [50]. Similarly transverse and sigmoid sinus thrombosis can also lead to severe complications [19].

### 4.2. Neurological Complications

#### 4.2.1. Facial Nerve Injury

The risk of facial nerve dysfunction after VS surgery cannot be entirely eliminated. A significant incidence of transient facial nerve dysfunction is still present after VS surgical removal [22, 51]. The type of surgical approach and tumor size are the main factors significantly affecting postoperative facial nerve function [52–54]. The anatomical facial nerve preservation rate has been reported to be 80–90% [55–59]. The preservation of facial nerve function has been reported to be 70–80% for tumors greater than 3 cm in diameter, removed either by the retrosigmoid [18, 43, 60] or by translabyrinthine approaches [55–59]. The HB evaluation of facial nerve function can be modified to “excellent” (HB grade 1/2), “intermediate” (HB grade 3/4), or “poor” (HB grade 5/6). The reported preservation rate of “excellent” function after the removal of large tumors is 42–52.6% [55–59].

Surgical techniques for preserving facial nerve function include the early identification of the root entry/exit zone and maximum caution during removal of the intrameatal tumor portion. If facial nerve function does not return after several months or the return of the function is not expected the cross-anastomosis is provided as the best measure and the most widely used technique for reanimation of facial nerve paralysis with no proximal stump [61–66]. Many different types of cross-anastomosis of damaged facial nerves have been described (accessory, phrenic, and glossopharyngeal nerves). The advantages of CN XII–CN VII anastomosis in general are improved facial tone with better cosmetic result, protection of the eye, intentional facial movements controlled by the tongue, and movements associated with physiological function of the tongue. The disadvantages are hemiatrophy of the tongue, mass movement of the face, and, in some instances, facial hypertonia [61]. This technique has been modified by May and subsequently Darrouzet who support side to end CN XII–CN VII anastomosis to preserve tongue function [21, 67]. We found this technique to be an excellent option for cases with an absence of the proximal stump of the CN VII. All patients were able to gain normal tonus of the face with voluntary movements and only transient dysarthria.

#### 4.2.2. Injury of Other Cranial Nerves

The facial numbness caused by trigeminal paresis (incidence 0–4.7%) [16, 43, 57, 68, 69] and the dysfunction of the abducens nerve are very rare complications of VS surgery on large tumors.

The lower cranial nerves can be injured during removal of large vestibular schwannomas, which should impinge on jugular foramen contents and CN IX, X, and XI. Acute lower cranial nerve deficits could result in dysphagia and aspiration. In some cases, it is necessary to provide nutrition through a nasogastric feeding tube or to prevent the risk of aspiration. Reeducation and rehabilitation of swallowing techniques and manoeuvres are crucial for management. In cases with severe problems and risk of aspiration pneumonia, tracheostomy and percutaneous endoscopic gastrostomy can be an option. In our study we encountered only transitory lower cranial nerve injury.

#### 4.2.3. Disordered Vestibular Compensation

Balance problems are a common complication of VS pre- as well as postoperatively [70–72]. Preoperative vertigo can be caused by a peripheral lesion (inner ear or neural origin); however, it can also have a central (cerebellar) origin [73]. Small tumors are more commonly associated with vertigo of peripheral origin because of missing deafferentation [74, 75], whereas large tumors, especially those that are slow-growing, will cause compression of the cerebellum or brainstem [74] and therefore central origin of vertigo. The preoperative diagnosis of a vestibular lesion is important for predicting the postoperative compensation outcome. Patients with a central lesion would be expected to have prolonged compensation [76], whereas in patients with hypofunction of the inner ear the development of compensation prior to surgery would be expected. It can be concluded that the severity of the patient's vestibular symptomatology is related to the level of residual vestibular function present; therefore, prehabituation (preoperative chemical labyrinthectomy with intratympanic application of gentamicin followed by vestibular rehabilitation) to improve vestibular compensation seems to be a promising method to alleviate postoperative problems [77]. Postoperative vertigo is in general caused by acute deafferentation originating from the transection of vestibular nerves. It has been reported in the majority of cases; however, it has a tendency to gradually improve over time [72, 78]. The prolonged postoperative vertigo, or disbalance, can be attributed to several factors: brainstem or cerebellum injuries, incorrect or insufficient rehabilitation, orthopedic and neurological factors, impairment of vision, anxiety and depression, and the persistence of the vestibular nerve [79, 80]. Nonaka et al. [14] described the influence of different surgical approaches on prolonged vertigo (in 12% of patients treated with the retrosigmoid approach, in 11.7% of patients with the middle fossa approach, and in 5% of patients undergoing the translabyrinthine approach). Overall incidences of persisting postoperative vertigo and disequilibrium in VS microsurgery have ranged from 1% to as high as 30% [16, 69, 81]. In our previous study [34], the patient's age was identified as the only important factor associated with disordered vestibular compensation following retrosigmoid VS microsurgery. Thus, new methods including prehabituation and biofeedback seem to be a logical step of outcome improvement [72, 77]. Intraoperative avoidance of cerebellar injury is crucial for the prevention of disordered vestibular compensation.

## 5. Conclusions

Despite the atypically high proportion of large tumors in this series, our rates of VS microsurgery complications are comparable with the reviewed literature, with the exception of the lateral CSF leak. Overall, during the last decades the rate of VS microsurgery complications has drastically decreased. However, the information about possible complications should be clearly given to the patient when the benefit/risk ratio is to be evaluated at the time of treatment decision. It can be concluded that most complications are the consequence of inadequate surgical maneuvers, with vascular complications carrying the most significant rate of severe morbidity and potential mortality. Appropriate selection of cases, meticulous surgical technique, and careful postoperative care are crucial to lower the rate of all complications of vestibular schwannoma microsurgery.

## Figures and Tables

**Table 1:** Complications of vestibular schwannoma microsurgery.

Type of complication	Number of patients	%
CN VII discontinuity	31	10%
Permanent CN V dysfunction	3	1%
Transient CN V dysfunction	7	2%
CN VI palsy	1	0.3%
Transient CN IX–XI dysfunction	20	6%
Disordered vestibular compensation	43	13%
Lateral variant of CSF leak	208	62.5%
Medial variant of CSF leak	2	0.6%
Headache	29	9%
Intracranial hemorrhage	12	4%
Epidural hematomas	3	1%
Mortality	3	3%

CN: cranial nerve.

**Table 2:** Facial nerve function assessed immediately after surgery and at the last follow up.

CN VII function (House-Brackmann)	1	2	3	4	5	6
Nm. immediately/last follow-up	140/195	35/19	64/89	32/9	25/6	23/0
% immediately/last follow-up	44/61	11/6	20/28	10/3	8/2	7/0
